# Supplementary material for: Lightweight deep learning for real-time road distress detection on mobile devices
Source: Nat Commun. 2025 May 6;16:4212. doi: 10.1038/s41467-025-59516-5 (PMC12055982; doi:10.1038/s41467-025-59516-5)
Supplement: Supplementary file 1 — Supplementary Information [file 41467_2025_59516_MOESM1_ESM.pdf]

Supplementary Information

Lightweight Deep Learning for Real-Time Road Distress Detection on Mobile Devices

Yuanyuan Hu [1], Ning Chen [2], Yue Hou [3,*], Xingshi Lin [4], Baohong Jing [5], Pengfei Liu [1,*]

[1] *Institute of Highway Engineering, RWTH Aachen University, 52074 Aachen, Germany*

[2] *Beijing Key Laboratory of Traffic Engineering, Beijing University of Technology, 100124 Beijing, China*

[3] *Department of Civil Engineering, Faculty of Science and Engineering, Swansea University, SA1 8EN Swansea, UK*

[4] *Fujian Yongzheng Construction Quality Inspection CO. LTD, 350012 Fuzhou, China*

[5] *Qingdao Yicheng Sichuang Link of Things Technology Co. LTD, 266555 Qingdao, China*

These authors contributed equally: Yuanyuan Hu, Ning Chen.

** Corresponding author. E-mail address: liu@isac.rwth-aachen.de (Pengfei Liu); yue.hou@swansea.ac.uk (Yue Hou).*

Supplementary Methods

YOLO Application Dataset Preparation and Model Conversion

The dataset used to train YOLOv8[1] consists of a combination of MR-captured images by the authors and open-source RDD2022[2] data, totaling 1,800 annotated samples. To ensure robustness across varying user perspectives, MR images were collected from individuals of different heights to simulate real-world variations in user positioning. This multi-height data collection approach accounts for the significant differences in viewing angles that occur in practical MR deployment scenarios, enhancing the model's ability to generalize across diverse user heights and device placements.

The dataset encompasses four primary distress categories consistent with the main study: longitudinal cracks, transverse cracks, alligator cracks, and broken road markings. Each image was meticulously annotated using bounding boxes to precisely localize distress instances, with annotations verified by transportation engineers to ensure accuracy and consistency with industry standards.

For the YOLOv8 model, the conversion process from the original training format to deployment-ready TFLite involved multiple technical steps. Initially, the trained YOLOv8 model was exported from PyTorch format (.pt) to ONNX (Open Neural Network Exchange) format using the export functionality built into Ultralytics' YOLOv8[1] implementation. Dynamic input sizing and model simplification were enabled to maintain flexibility while

reducing complexity. Subsequently, the ONNX model was converted to TensorFlow format (.pb) using the ONNX-TensorFlow conversion tool, which transformed the model architecture and weights while preserving network topology and numerical precision. The TensorFlow model then underwent optimization through TensorFlow's graph optimization tools. Finally, the optimized model was converted to TFLite format using the TensorFlow Lite Converter with quantization applied to reduce model size and improve inference speed while maintaining detection accuracy. Despite these comprehensive optimization efforts, the final YOLOv8 TFLite model remained computationally intensive due to its inherent architectural complexity, resulting in the longer processing times observed during comparative testing with the MobiLiteNet-optimized MobileNet V2 model.

Supplementary Discussion

Image Comparison of MR-Based Road Distress Detection Applications

The supplementary images provide visual evidence of the field validation conducted in Aachen, Germany, demonstrating the practical implementation of the MR-based road distress detection systems developed in this study.

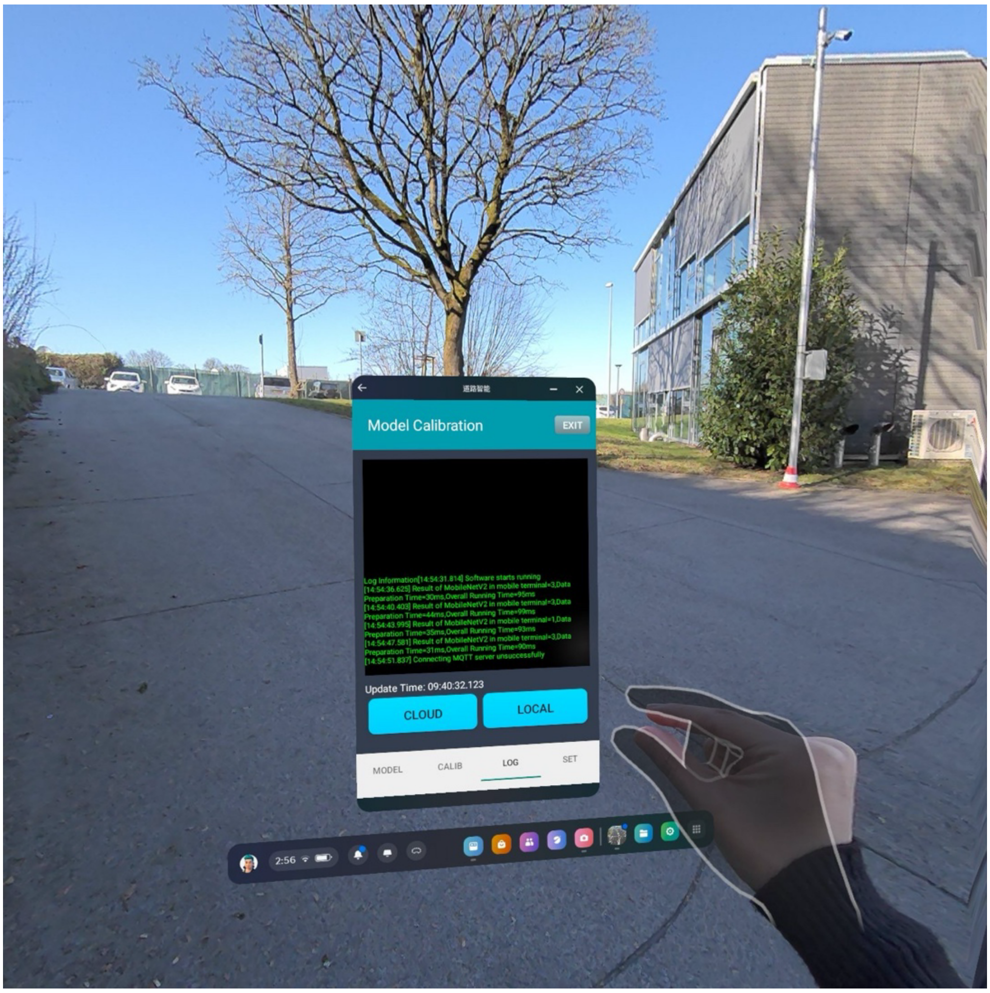

Supplementary Fig. 1. MobiLiteNet-Based RoadIntelligent Application

Supplementary Fig. 1 displays the RoadIntelligent application utilizing the MobiLiteNet-optimized MobileNet V2 model during calibration and field testing. The system log information (displayed in green text) shows the

model initialization and execution process, with processing times significantly lower than the YOLO implementation. The application's rapid processing capabilities enable genuine real-time detection, validating the effectiveness of the optimization techniques employed in the MobiLiteNet framework.

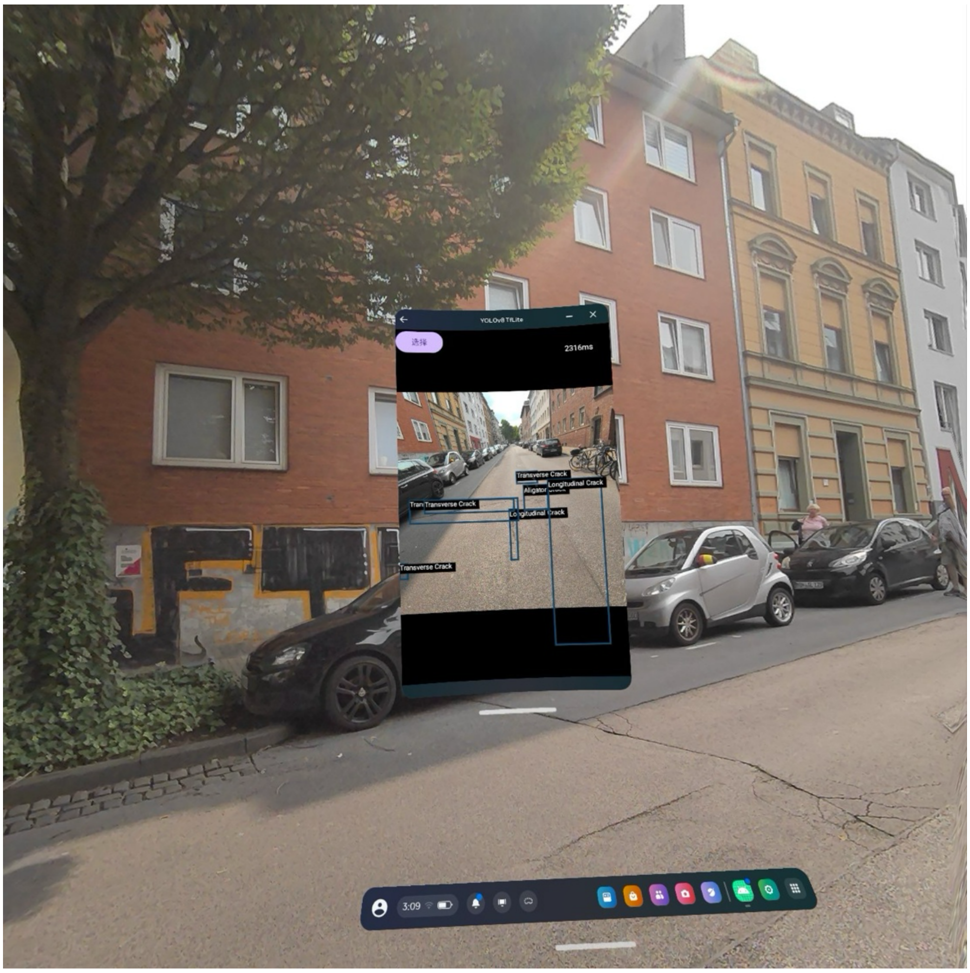

Supplementary Fig. 2. YOLO Application for Road Distress Detection

Supplementary Fig. 2 shows the YOLO application deployed on MR devices during field testing. The application successfully identifies multiple road distresses including longitudinal cracks and transverse cracks, as indicated by the bounding boxes and classification labels. The processing time (2316ms) is displayed in the upper right corner, demonstrating the computational intensity of the YOLO-based implementation. Despite its high detection accuracy, the significant processing time illustrates the challenges of deploying complex detection models on resource-constrained MR devices for real-time applications.

REFERENCES

1. Jocher, G., Chaurasia, A., Qiu, J. & Ultralytics. YOLO by Ultralytics. GitHub repository, https://github.com/ultralytics/ultralytics (2023).

2. Arya, D., et al. RDD2022 - The multi-national Road Damage Dataset released through CRDDC'2022. figshare. Dataset (2022).
