# Supplementary material for: Lightweight deep learning for real-time road distress detection on mobile devices
Source: Nat Commun. 2025 May 6;16:4212. doi: 10.1038/s41467-025-59516-5 (PMC12055982; doi:10.1038/s41467-025-59516-5)
Supplement: Supplementary file 2 — Description Of Additional Supplementary File [file 41467_2025_59516_MOESM2_ESM.pdf]

Description of Additional supplementary files

Supplementary Movie 1: RoadIntelligent Application on MR device.

Supplementary Movie 2: RoadIntelligent Application on smartphone.
